# Hospitalizations due to diabetes mellitus from January/2008 to February/2015 in Brazil – a datasus based report

**DOI:** 10.1186/1758-5996-7-S1-A193

**Published:** 2015-11-11

**Authors:** Erika Cesar de Oliveira Naliato, Ramon Gabriel Souza de Oliveira, Victor Luiz Sepúlveda Rey

**Affiliations:** 1 Centro de Estudos Ricardo A T Castilho-Associação Médica de Teresópolis, Teresópolis, Brazil

## Background

Brazil has a high prevalence of diabetes mellitus (DM) and this disease is known to increase morbidity through the development of micro- and macrovascular complications, as well as acute events, such as infections. Both the disease and its complications may generate hospitalization.

## Aim

To study hospitalization due to DM from January/2008 to February/2015, using information available at the DATASUS data bank.

## Materials and methods

Data from the Hospital Information System of SUS (Single Health System-Brazil) was accessed in the DATASUS data bank (Ministry of Health-Brazil) to allow the study of the hospitalization due to DM and also its association with the following diagnosis of the CID-10 morbidity list: renal insufficiency, cataract, glaucoma, retinal detachment, amaurosis, subnormal vision, disturbances peripheral nerves, cerebrovascular accidents, arterial hypertension, myocardium infarct, atherosclerosis, and infections.

## Results

From January/2008 to February/2015, 591,867,160 hospitalizations due to DM were identified in Brazil. In this period, 1,631,428 of the diabetics (0.28%) that were hospitalized had renal insufficiency. Regarding the association between DM and ocular diseases, 1,267,294 of the diabetics had cataract (0.21%), 1,105,881 presented retinal detachments (0.19%), 1.035,520 had glaucoma (0.18%) and 1.013,679 had amaurosis or subnormal vision (0.17%). A disturbance of the peripheral nerves and plexus was associated with DM in 1,167,256 patients (0.20%). Cerebrovascular accident was present as an associated diagnosis in 2.070,171 hospitalized diabetics (0.35%), arterial hypertension in 1,656.050 (0.28%), acute myocardium infarct in 1,583,107 and atherosclerosis in 1,115,565 (0.19%). Pneumonia, upper airway and skin and subcutaneous infections were present in 6,720,865 diabetics (1.14%).

## Conclusion

In the present study using the DATASUS system, the most common comorbidity present in subjects hospitalized due to DM was infection. However, the authors did not consider the DATASUS data bank the ideal source for study of the association between DM and complications since, once the DM is chosen as the diagnosis, the other CID-10 are not concomitantly annotated in a routine basis and consequently are under-estimated in the system.

